# Some considerations for analyzing biodiversity using integrative metagenomics and gene networks

**DOI:** 10.1186/1745-6150-5-47

**Published:** 2010-07-30

**Authors:** Lucie Bittner, Sébastien Halary, Claude Payri, Corinne Cruaud, Bruno de Reviers, Philippe Lopez, Eric Bapteste

**Affiliations:** 1 UMR CNRS 7138 Systématique, Adaptation, Evolution, Muséum National d'Histoire Naturelle, Paris, France; 2 UMR CNRS 7138 Systématique, Adaptation, Evolution, Université Pierre et Marie Curie, Paris, France; 3 UR227, IRD-BPA5, Nouméa, Nouvelle-Calédonie, France; 4 Genoscope, Centre National de Séquençage, Evry, France

## Abstract

**Background:**

Improving knowledge of biodiversity will benefit conservation biology, enhance bioremediation studies, and could lead to new medical treatments. However there is no standard approach to estimate and to compare the diversity of different environments, or to study its past, and possibly, future evolution.

**Presentation of the hypothesis:**

We argue that there are two conditions for significant progress in the identification and quantification of biodiversity. First, integrative metagenomic studies - aiming at the simultaneous examination (or even better at the integration) of observations about the elements, functions and evolutionary processes captured by the massive sequencing of multiple markers - should be preferred over DNA barcoding projects and over metagenomic projects based on a single marker. Second, such metagenomic data should be studied with novel inclusive network-based approaches, designed to draw inferences both on the many units and on the many processes present in the environments.

**Testing the hypothesis:**

We reached these conclusions through a comparison of the theoretical foundations of two molecular approaches seeking to assess biodiversity: metagenomics (mostly used on prokaryotes and protists) and DNA barcoding (mostly used on multicellular eukaryotes), and by pragmatic considerations of the issues caused by the 'species problem' in biodiversity studies.

**Implications of the hypothesis:**

Evolutionary gene networks reduce the risk of producing biodiversity estimates with limited explanatory power, biased either by unequal rates of LGT, or difficult to interpret due to (practical) problems caused by type I and type II grey zones. Moreover, these networks would easily accommodate additional (meta)transcriptomic and (meta)proteomic data.

**Reviewers:**

This article was reviewed by Pr. William Martin, Dr. David Williams (nominated by Pr. J Peter Gogarten) & Dr. James McInerney (nominated by Pr. John Logsdon).

## Background

### Studying biodiversity

Improving knowledge of biodiversity will benefit conservation biology[1], enhance bioremediation studies[2], and could lead to new medical treatments[3]. However there is no standard approach to estimate and to compare the diversity of different environments, or to study its past, and possibly, future evolution. Part of the problem is that analyses of biodiversity require both a clear definition of the term biodiversity, e.g. what are the relevant units of biodiversity considered, and a consensus on the methods relevant to quantify these units. However, biodiversity is a complex notion, which raises multiple questions that can be addressed from distinct perspectives[4]. First, compositional[5] or element-based accounts of biodiversity[6] can inquire "What is there?". Then, scientists describe the variety of life forms (or bio-specifics[6]) present in an ecosystem, such as the genes, organisms, species, clades, and communities. Second, functional[5] account of biodiversity[6] can examine what these elements are doing: "What is happening out there?". Here, the focus shifts towards the many functions fulfilled in the ecosystem. Third, a process-based perspective can address evolutionary questions, such as: "How did these elements and functions evolve?". Studies thus concentrate on how diversity is generated and sustained by processes, such as mutation, recombination, lateral gene transfer, ecological pressures, and the like. These profoundly different viewpoints lead to distinct estimates of natural diversity, highlighting most important issues regarding the identification and systematisation of biodiversity.

The element-based perspective typically offers various measures of biodiversity reflecting either the number and/or the phylogenetic diversity of bio-specifics, or their complementarity in various environments[7,8]. It resulted in multiple biodiversity indices serving different purposes[9]. In particular, Faith[6,10] suggested to account for the whole hierarchy of bio- specifics (from lower-level genetic units to higher clades) and for within species diversity. However, the inventory of species - and the inventory of elements in general - has been criticized by philosophers for providing a static rather than a dynamic account of biodiversity, neglecting biological processes[11]. Element-based approaches cleave the ecosystem into known static bits and pieces[12,13], while deeper analyses of natural variation require a more integrated and dynamic understanding of the processes affecting the ecosystem as a whole[14]. Then, relevant estimates of biodiversity cannot only be based on indices derived from lists of elements, they should also quantify the likelihood that a given ecosystem, as a whole, will continue producing natural diversity[15]. Problematically, details of the functional integration of the many elements of an ecosystem, and of the evolutionary processes affecting their diversity, are almost always largely unknown. Hence, practical (and quantitative) process-based studies of biodiversity have only recently got under way.

Since no single perspective provides a satisfactory account of biodiversity, confronting multiple approaches can suggest possible improvements in biodiversity studies. Here we contrast two approaches that are among the many tools used for studying biodiversity: microbial metagenomics[16] and DNA barcoding[17]. Based on their divergences, we propose an integrative approach aiming at the simultaneous examination (or even better at the integration) of the elements-based, function-based and process-based perspectives in biodiversity studies, thanks to massive sequencing of multiple environmental markers. We argue that it should be preferred over DNA barcoding and metagenomic studies based on a single marker for a variety of organisms for which the definition of species is ambiguous (prokaryotes and eukaryotes alike). Finally, we discuss how network-based analyses of such molecular datasets could benefit biodiversity studies.

### The two diverging tracks of microbial metagenomics and DNA barcoding Species is not the relevant unit in metagenomic studies

Both studies of microbial metagenomics and DNA barcoding initially adopted an element-based perspective of biodiversity[18]. They focused on the identification and the quantification of compositional units rather than on the processes sustaining the diversity (with, later, major exceptions concerning metagenomics). Interestingly however, microbial metagenomics and DNA barcoding differed in their units of interest.

Microbial metagenomics is an assumed gene-centric perspective, that consists of the direct sequencing of environmental DNA[19]. It uses either one marker (sequenced at very high depth)[20] or many (generally randomly amplified)[21] to analyze phylotypes and/or functional categories[22]. Phylotypes are groups of homologous sequences (usually the 16 S rRNA) whose members share more than a given percentage of similarity (e.g. over 99% of sequence identity). They are defined to assign environmental sequences[23] to a taxon of reference, by BLASTing[24] the phylotypes against databases of identified taxa[25,26]. This 'taxonomic' assignation highly depends on the gene's conservation across taxa, the depth of the taxonomic sampling in the databases, the taxon richness and evenness in the environmental sample, the sequence read length and the impact of lateral gene transfer (LGT) in the environment and in the reference database[27]. LGT is caused by processes such as transposition, transduction, and conjugation. LGT results in significant variations in the gene content of even closely related strains thriving in different environments[28]. Consequently, a single gene, even the 16 S, provides limited information about biodiversity[29-32], and does not necessarily allow reliable prediction of community metabolism, physiology, biochemistry and ecology[33]. Typically, different ecosystems that cannot be distinguished by their phylotypes can be distinguished by their functions[33].

Importantly, microbial metagenomicists acknowledge that phylotypes are defined *ad hoc *[27] in order to obtain discrete categories, usable in various calculations of diversity (such as rarefaction curves or Chao1 estimates[8,9]). Likewise, environmental sequences are binned into functional categories, based on BLAST searches against reference databases[23,34] (KEGG[35], Pfam[36], SEED[36]). Such practical units aim at sampling the total genetic diversity to expand our knowledge of the gene content, functional significance and genetic variability in natural communities[1,22,33]. A key point here is that microbial metagenomics does not use species as the standard unit to describe and to quantify biodiversity.

**Figure 1:**
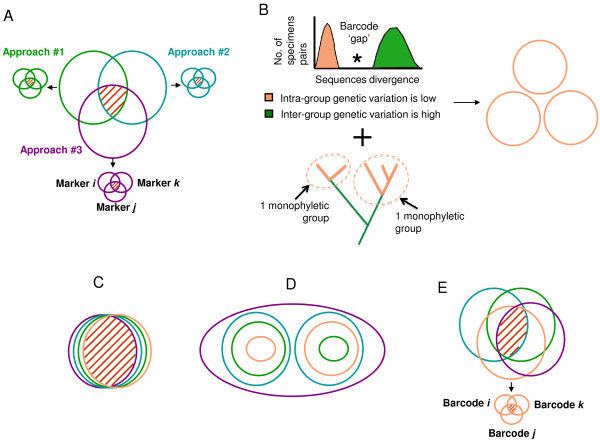
**Four remarkable situations when distinct species concepts are applied**. Each species concept groups a set of organisms, as members of a species taxon, as illustrated by a colored circle (purple for the phylogenetic species, green for the recombining or biological species, blue for the morphological species, pink for the barcode-based species). The overlap between groups is indicated by red dashes. A. In prokaryotes, the groups defined by the various species concepts are largely not nested. A unified species concept would be a poor descriptor of biodiversity: inter-approach pluralism is an issue for species definition. So is intra-approach pluralism, as indicated by smaller circles corresponding to the incongruent groups proposed by different markers, for a given species concept. B. Exploratory use of DNA barcoding to define groups of specimens belonging to a same species. On a histogram of p-distances frequencies, the identification of a barcode gap provides a threshold over which two specimens cannot belong to the same species. The monophyly of specimens falling in a same group can also be assessed. C. The ideal case: all the species concepts identify the same sets of organisms. Intra- and inter-approach pluralisms are not a problem. A unified species concept is a good descriptor of biodiversity D. Type I grey zone: the species concepts produce a series of nested groups. Ranking these groups is an issue. E. Type II grey zone: the species concepts produce partially non-nested groups. Inter- and intra-approach pluralism can be problematic. For cases D & E, pragmatic descriptors would be more accurate and informative about biodiversity than a unified species concept.

There are good reasons for this choice: species pose at least two major problems to microbial diversity studies. First, species raise the problem of inter-approach pluralism[37]. Microbiologists studying prokaryotes do not adhere to a single (unified) species concept[37]. Rather, they recognize different but equally legitimate rules (or 'species concepts') to group individual microbes as members of a given species taxon[37]. These rules rest on distinct criteria, many of which are based on different evolutionary and ecological processes, capturing diverse important features of microbial diversity[38,39]. Consequently, there is no guarantee that individual microbes should always fall into recognizable discrete groups, showing tight genotypic and phenotypic similarity as well as genetic connectivity[40-43]. For a given set of individuals in a given environment, this plurality of legitimate rules can and does produce a plurality of valid incompatible groups[44-48] (Figure 1A). However, if different species concepts are used to assess the diversity in different environments, estimates of the number and composition of species are not directly comparable.

Second, species raise the issue of intra-approach pluralism[37] in microbial biodiversity studies (Figure 1A). Even with a given species concept, one can sort the same organisms into conflicting classifications, depending on the empirical evidence at hand. In prokaryotes, this conflict in species definition is largely due to the prevalence of LGT. For instance, since recombination is usually limited to parts of a genome, the definition of recombining-species depends on which part of the genome is selected[46,49]. Likewise, given the frequency of LGT, and since genetic isolation occurs on a gene-by-gene basis in prokaryotes[49], different clusters of genes of an organism's genome legitimately support different -uncoupled- evolutionary histories[37]. It affects the identification of phylogenetic- species. Hence, in the absence of a unified species concept, counting species will always be problematic, method-, marker-, and possibly sample-dependent. Instead, more precise operational units could be used to capture genetic biodiversity at multiple levels, and ideally to reveal the evolutionary processes taking place in an environment[38,39].

### Species is the relevant unit in DNA barcoding studies

DNA barcoding contrasts with metagenomics. DNA barcoding is currently mostly used on multicellular eukaryotes but with the ambition of studying the entire diversity of life. It is a minimalistic strategy, that has aided taxonomic work and biodiversity studies, by sorting and clustering specimens collected in the field, contributing to species discovery by flagging genetically distinct lineages. Barcoders sequence 400-600 base pairs of a single molecular marker (or barcode) with a strict uniparental inheritance[17,50], such as the mitochondrial cytochrome c oxidase 1 gene[51], to divide life into such natural units. Comparative analyses of these barcodes can serve to define species boundaries (although it is not their only use), and to study genetic diversity within these species. Pairwise distances (usually p-distances) are inferred from the barcodes of all specimens under study. Under the assumption that pairs of sequences from a same species are more similar than pairs of sequences from different species[50], the presence of multiple species in a sample should produce a characteristic barcode-gap[50,52], separating sequences with low p-distances from sequences with high p-distances on an histogram of p-distances frequencies[52] (Figure 1B). Such graphs are used to compute a minimal p-distance over which two sequences do not belong to the same species. Alternative approaches use maximum likelihood-based methods, models of coalescence and speciation processes, to delineate groups of specimens belonging to a same species from barcode sequences[53,54]. In both cases, these barcode-based species are tentatively assigned to a known species by comparison with sequences from previously recognized taxa[55] to refine estimates of intra-specific genetic variation. In absence of matches with reference species, each group of specimens sharing very similar barcodes is identified and counted as a new species. Their monophyly on a representative gene tree is sometimes a further condition[50,56]. Finally, in DNA barcoding, when such groups are proposed, no additional genes are generally required to evaluate biodiversity. No functional analysis is achieved.

In what follows, we won't question DNA barcoding first objective (i.e. to assign unknown specimens to already recognized species, thanks to a DNA-library of named specimens). This approach has the potential to produce estimates of the genetic diversity within accepted species. Our claim will only concern DNA barcoding second - exploratory- goal (i.e. enhancing the discovery of new species, particularly in cryptic, microscopic and other organisms with complex or inaccessible morphology, considering that genetic-species could be delineated based on the analysis of the genetic distances between unassigned specimens, using the working hypothesis that inter-specific genetic distances should have greater values than intra-specific ones). More precisely, the fact that DNA barcoding and microbial metagenomics offer separate recipes to estimate biodiversity raises questions concerning which units and methods provide the most informative account of biodiversity using molecules.

## Presentation of the hypothesis

### The pragmatical road to integrative metagenomics

Differences in assessment of biodiversity by DNA barcoding and microbial metagenomics may be reasoned by contrasting their biological scope: microbial metagenomics mostly studies prokaryotes[16] (affected by LGT) and DNA barcoding has yet mostly studied eukaryotes[56] (resistant to LGT). Certainly, estimates of the diversity of elephants and the diversity of *E. coli *are pretty different questions. However, a deeper explanation of the differences between DNA barcoding and microbial metagenomics probably lies in their distinct underlying philosophies, with respect to the 'species problem'.

### Identifying species problems

Unlike microbial metagenomics, DNA barcoding implicitly endorses a unified species concept (USC). At first look, an USC offers a unique advantage: the possibility of unambiguous definitions of species, and thus of informative units that can be compared in biodiversity studies. The most popular USC is the general lineage concept [57,58] of de Queiroz[59]. In that theory, species correspond to separately evolving lineages of metapopulations. The various rules defining diverse species taxa (ecological, phylogenetic, biological, and so on) do not directly define the species boundaries. They only capture distinct secondary properties of the species, providing operational criteria that emphasize different processes responsible for some coherence between organisms. The species boundaries can however be derived by analyzing how the groups defined by these distinct secondary operational criteria overlap. In particular, if they all largely agree, the species is simply bounded by the intersection of the groups (Figure 1C). All the species members then share a common biochemistry, physiology, sexual behaviour, phylogeny, and ecology. In that ideal case, a group of organisms identified by DNA barcoding only provides a good proxy for the species boundaries, and a valuable measure of biodiversity. One might however wonder whether the different groups proposed by distinct secondary concepts should always largely intersect, and if not, whether counting species, using DNA barcoding groups as a proxy, provides meaningful estimates of biodiversity.

When different criteria support conflicting (or weakly overlapping) groups of organisms, a 'grey zone' appears[58]. For us, two sorts of grey zones (Figure 1D&1E) strongly confuse species-based estimates of biodiversity attempted by DNA barcoding. First, when the various secondary properties defining the groups arose at different times in the process of speciation, the groups that can be proposed will be typically nested (Type I grey zone, Figure 1D). Such compatible albeit incongruent groups have been very often reported in studies of eukaryotic diversity[60-63]. Second, the different criteria can define partly overlapping (e.g. not nested) sets of organisms (Type II grey zone, Figure 1E). Many biological processes lead to this result, such as incomplete lineage sorting associated with very rapid or recent speciations[61], introgression[51], hybridization and polyploïdy[62]. Differences in organellar and/or nuclear evolution also produce legitimate disagreement between groups inferred using these two sources of characters, when nuclear and organellar genomes have distinct coalescence times [64], effective population sizes[65,66], or when biparental inheritance[67,68] and heteroplasmy[69] of the organelles is undetected. Moreover, in organisms and lineages with variable frequencies of sexual reproduction[63] and clonality[62], several combinations of the migration rates[70], ranges and modes of dispersal[63] equally generate non-nested genetic, morphological, ecological, and phylogenetic groups. Type II grey zones were notably reported in 17% of the 89 studies conducted using phylogenetic and non- phylogenetic concepts to analyze the diversity of multicellular eukaryotes (e.g. grass, fungi, and metazoans)[60]. Importantly, no such estimate has yet been compiled for unicellular eukaryotes: the level of incongruence between groups used to define the species boundaries may be comparable to that of multicellular eukaryotes, but the correspondence between DNA sequence clusters, ecotypes and morphospecies is still largely unknown for protists [61-63,70,71].

### Dealing with species problems

Type I grey zones confronts DNA barcoding studies of biodiversity to serious practical issues. Identifying *bona fide *species in such a continuum of groups within groups poses the famous ranking problem, e.g. the need for decision criteria to assign monophyletic lineages to distinct taxonomical ranks. Unfortunately, the USC, that justifies DNA barcoding approach, does not offer any additional operational criteria to decide where the species ends and starts[59]. A comparable conundrum was for thinstance met when biologists attempted to discriminate species from varieties in the XIX century. When no real boundary but only arbitrary differences existed between the two, Darwin compared the ranking problem to defining the indefinable[59]. He concluded that we, not nature, draw divisions - and identify species - for pragmatic reasons.

It is thus important to wonder whether DNA barcoding operates pragmatic divisions, relevant for biodiversity analyses, and in particular defines species so they can be compared between studies. Some considerations suggest that it is unfortunately not the case. First, there is no universal barcode[72-76]: different markers must be used for different organisms. Thus, biodiversity studies cannot always compare like to like. Second, not all datasets present a nice barcode gap, which affects the delineation of groups. Moreover many artefacts produce barcode gaps, hindering the identification of *bona fide *species[76,77]. For instance, the threshold over which two sequences are considered too distant to belong to the same species is directly affected by the sampling effort[61,78], and by the biology of the organisms under study. In particular, issues of hidden paralogy, presence of nuclear copies of mitochondrial genes[79], cases of biparental inheritance[67,68], variable coalescence times[80,81], unequal molecular evolutionary rates[82] and migration rates[77] can bias groups definitions. Unlike phylotypes in microbial metagenomics analyses, units by which biodiversity is counted are not held constant in independent DNA barcoding studies[83]. Typically, the pairwise distance corresponding to species membership changes with the sampling effort and between samples [50]. Thus direct quantitative and qualitative comparisons of biodiversity estimates are not feasible, which seriously limits the evaluation of the extent and of the evolution of biodiversity, across environments and over time.

Type II grey zones, which result from the genuine identification of different types of lineages, caused by distinct processes pose a different practical puzzle. It extends the problem of inter-approach pluralism to DNA barcoding analyses. The USC leads to the identification of species that are actually heterogeneous in terms of biological processes and structures. For that reason, species have limited explanatory power[37]. The groups proposed by DNA barcoding approaches no longer convey much information about the ecology, physiology, etc. of the species and about the processes (migration, interbreeding, adaptations, duplications, transpositions, etc.) sustaining these properties. Biodiversity measures based on a single feature will unfortunately provide an unrepresentative estimate. The larger the type II grey zone, the bigger the issue. Hence, it might appear more pragmatic to devise additional units with explanatory and predictive utility, for instance interbreeding groups, ecological groups, smallest phylogenetic groups worthy of recognition, to assess what processes crucially maintain biodiversity[60,84,85]. This solution seems even more relevant if, for type II grey zone, the use of different barcodes produces different groups (Figure 1E). Such cases of intra- approach pluralism may further distort biodiversity estimates in DNA barcoding studies, since disagreement between barcodes is not unexpected[61,71,86].

### Corallinales as a case-study

Corallinales are a worldwide distributed order of red algae with calcite in their cell walls. They often reproduce asexually via thallus fragmentation, direct asexual spores, produce unattached rhodoliths and grow on every favourable substratum (i.e. shells, drifting woods, drifting algae). Delineating species within Corallinales is difficult, because environmental conditions (such as the strength of currents) impact their morphology and collected specimens are generally sterile. DNA barcoding approaches were recently applied to small, geographically restricted, datasets of Corallinales[87,88]. It was reported that intra- specific genetic diversity was at least twice smaller than the inter-specific genetic diversity between already recognized morpho-species, suggesting that DNA barcode studies could help discovering new species of Corallinales. However, analyses of additional sequences from two markers (240 mitochondrial CO1 genes and 495 plastidial *psb*A genes), mainly from South Pacific Ocean (Additional file 1) unravelled both type I and type II grey zone related issues for these taxa.

**Figure 2:**
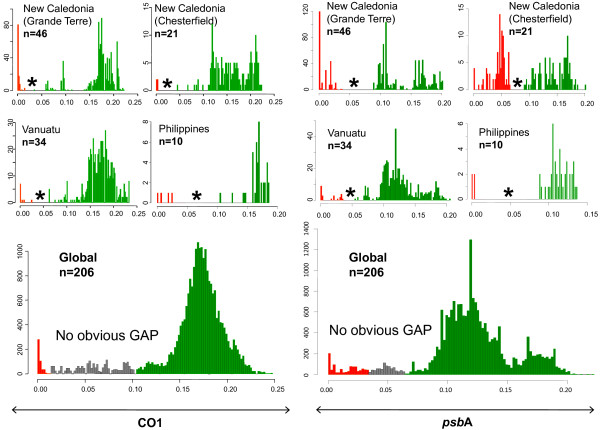
**Histograms of the frequency of p-distances for CO1 and *psb*A in a Corallinales Dataset**. A. Results for the CO1 dataset: the horizontal axis represents the pairwise sequence divergence (p-distances) for the specimens of a given class of frequency; the vertical axis corresponds to the number of pairs of specimens of each class. 'n' indicates the number of specimens sampled for a given locality. Barcode gaps are indicated by a star. Inferred interspecific distances are reported in green, inferred intraspecific distances are reported in red. B. Results for the *psb*A dataset. Same legend. On the global sampling, no barcode gap can be defined. Several discontinuities exist in the distribution, as represented by the grey area. When more data are included (data not shown), the barcode gap disappears.

For 206 specimens sharing these two markers, BCG[78] and MYC[53] methods proposed inconsistent method-, locality- and gene-dependent estimates of the number of Corallinales species present in the dataset. Methodological biases and artefacts (e.g. the use of an incorrect ultrametric tree in the MYC approach or of a wrong model of evolution) can for sure explain some of the disagreement between methods (inter-approach pluralism). Yet, even for a given method the two markers generally returned incompatible estimates (Table 1). The closest assessments between CO1 and *psb*A presented an average of 45% of groups with different specimen contents. This intra-approach pluralism is problematic because it was impossible to determine whether and which of these incompatible groups may correspond to a unified 'species'. Each group had a lower degree of genetic diversity than that reported as *bona fide *intra-specific distance in previous studies[87-89]. All showed a comparable coherence in terms of monophyly and morphology, and a similar lack of geographical coherence (data not shown). Partitioning the dataset by sampling sites also had a dramatic effect on biodiversity analyses (Table 2). For both markers, histograms of p-distances comprising the entire dataset showed no clear gap, while every site specific sub-sample presented a gap, seemingly defining an unambiguous limit for intra- and inter- genetic diversity (Figure 2). However, the genetic distances inferred from each site to define a species were highly variable. Problematically, between localities, some inter-specific distances overlapped with intra-specific distances (type I grey zone), and sometimes conflicted (type II grey zone)(Table 2). No standard threshold to define Corallinales species with CO1 or *psb*A could be proposed.

**Table 1:** Biodiversity estimates for each method and marker

gene\methods	BCG lower limit (nj tree)	BCG higher limit (nj tree)	MYC (UPGMA tree)
206 sequences of CO1 without EM	**129 **ESUs *i *= 0,0087	**37 ESUs** *i *= 0,137	**121 **ESUs (117-129)

206 sequences of CO1 with EM (HKY85)	**130 **ESUs *i *= 0,01	**38 ESUs** *i *= 0,259	**128 **ESUs (118-130)

206 sequences of CO1 with EM (GTR+I+G)	**55 **ESUs *i *= 0,620	**64 ESUs** *i *= 0,531	**128 **ESUs (125-129)

206 sequences of *psb*A without EM	**52 ESUs** *i *= 0,066	**52 ESUs** *i *= 0,071	**90 **ESUs (74-101)

206 sequences of *psb*A with EM (HKY85)	**54 ESUs** *i *= 0,075	**11 ESUs** *i *= 0,224	**91 ESUs** (61-94)

206 sequences of *psb*A with EM (GTR+I+G)	**55 ESUs** *i *= 0,081	**63 ESUs** *i *= 0,047	**81 ESUs** (35-100)

Each column corresponds to the results of a given method. Each line was inferred with specific settings and evolutionary models (EM). Uncorrected p-distances were calculated using MEGA 4.1[112]. Parameters for two evolutionary models (HKY85; GTR with a gamma distribution splitting into 4 categories - GTR+I+G4) were calculated using PALM http://palm.iis.sinica.edu.tw/index.html and MrModeltest version 2.2[113], and then used to calculate corrected p-distances using PAUP* version 4b10[114]. NJ (Neighbor-Joining) and UPGMA trees were built using PAUP* version 4b10, considering no evolution model and then a HKY85, and finally a GTR+I+G model. A 'relative time from branching rate' was determined with the UPGMA trees using the GENIE v3.0 software[115]. Since it was not possible to identify a barcode gap unambigously, we defined a range of BCG estimates, based on the histogram of p-distances. The first empty class of frequency defined the lower limit for the intraspecific distance (BCG lower limit). The higher limit of the intraspecific distance (BCG higher limit) was defined as the left bound of the 95% confidence interval of the Normal distribution followed by the histograms of p-distances. The number of estimated monophyletic groups corresponding to species (ESUs) is indicated in bold. Values of *i *correspond to the upper bound estimated for the intraspecific pairwise-distance for the BCG methods. For the MYC method, a range of estimated ESUs is given into rounded bracket (confidence interval of 95%).

**Table 2:** Inferred intra and interspecific pairwise-distances for CO1 and *psb*A by sampling locality.

	n =	CO1 sequences identity average	CO1 intra ESUs variation	CO1 inter ESUs variation	*psbA* sequences identity average	*psbA* intra ESUs variation	*psbA* inter ESUs variation
**Fiji**	56	84.87%	0-3 bp *i < 0,0065*	**> 11 bp** **j >0,023**	89.64%	**0-39 bp** ***i *< 0,071** **(£)**	> 45 bp j >0,083

**New Caledonia**	46	84.73%	0-7 bp *i < 0,015*	> 16 bp j >0,034	89.02%	**0-20 bp** ***i *< 0,036**	> 48 bp j >0,088

**Vanuatu**	34	83.19%	**0-12 bp** ***i *< 0,026** **(£)**	> 29 bp j >0,062	87.93%	**0-19 bp** ***i *< 0,035**	**> 30 bp** **j >0,015** **(£)**

**Chesterfield**	21	84.10%	0-1 bp *i < 0,0021*	> 21 bp j >0,045	89.94%	**0-35 bp** ***i *< 0,064**	> 49 bp j >0,090

**Europe**	14	84.73%	0-8 bp *i < 0,017* **(£)**	> 46 bp j >0,099	89.42%	0-1 bp *i < 0,0018*	> 22 bp j >0,040 **(£)**

**Morea (French Polynesia)**	12	84.05%	0-1 bp *i < 0,0021*	> 37 bp j >0,080	88.46%	**0-19 bp** ***i *< 0,035**	> 48 bp j >0,088

**Philippines**	10	84.79%	**0-12 bp** ***i *< 0,026** **(£)**	> 48 bp j >0,104	89.60%	0-2 bp *i < 0,0036*	> 48 bp j >0,088

**Caribbean**	6	87.58%	0-7 bp *i < 0,015*	> 53 bp j >0,114	90.40%	0-3 bp *i < 0,0055*	> 42 bp j >0,077

**Indonesia**	4	87.42%	1 bp *i < 0,0021*	> 66 bp j >0,143	89.30%	2 bp *i < 0,0036*	> 64 bp j >0,118

**Global**	206	83.17%	0-4 bp *i < 0,0087*	> 7 bp j >0,015	88.15%	0-36 bp *i < 0,066*	> 37 bp j >0,068

For each locality, for n specimens, the table presents the average sequence identity, the inferred intraspecific (*i*) and interspecific (j) distances, without EM, and their corresponding variation in number of base pairs for the identified ESUs. Cells in bold indicates when intra- specific and inter-specific distances conflict, for a given marker, between different sites. Cells with a **(£) **indicates when intra-specific and inter-specific distances conflict, for a given marker, between a local site and the genetic threshold based on the global sampling.

**Figure 3:**
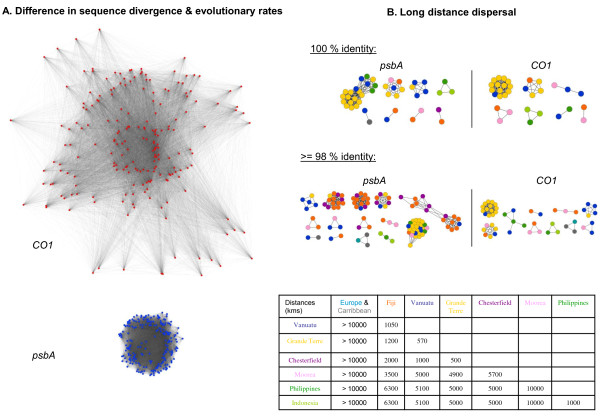
**Gene networks of CO1 and psbA datasets**. A. Sequence diversity of CO1 (in red) and *psbA *(in blue) datasets for the same 206 specimens represented by gene homology networks, using the same scale and the same parameters for display. Nodes are sequences, and edges lengths are roughly proportional to the percentage of sequence identity between sequences. Closer sequences are more identical. CO1 displays more genetic diversity than *psbA*, thus has evolved faster in these specimens. B. Network- based phylogeographic analysis of CO1 and *psbA *sequences only showing sequences sharing 100% identical sequences but found in distinct geographical sites. Same networks for sequences presenting over 98% of identity. Nodes are sequences, colored according to their geographical origin: orange for Fiji; yellow for New Caledonia - 'Grande Terre'; dark blue for Vanuatu; purple for New Caledonia - Chesterfield; sky-blue for Europe; pink for French Polynesia; dark green for Philippines; grey for the Caribbean; light green for Indonesia. The colour coded table indicates the corresponding distances between each pair of sites. The sequences with the highest proportion of identical matches are displayed closer in the graph.

Importantly, these inconsistent estimates can be explained by different evolutionary processes, with opposite influences, that sustain Corallinales biodiversity. On the one hand asexual reproduction and somaclonal mutation tend to produce divergent lineages and should produce congruent groups between markers; on the other hand, many other processes tend to mix genomes and should produce incongruent groups between markers. First, CO1 and *psb*A had different rates of evolution (Figure 3A). The fact that more species were generally detected with CO1 than with *psb*A, although these two markers had equally resolved phylogenies (41,6% of nodes with a support >80% for CO1 and 37,7% of such nodes for *psb*A), could be due to the faster evolutionary rate of mitochondrial DNA compared to plastidial DNA.

Second, the mutually incompatible groupings proposed by these markers could reflect lineage sorting, as illustrated in previous studies on seaweeds[90]. Since CO1 and *psb*A maximum likelihood trees (reconstructed with a GTR+I+G4 model, 1000 bootstrap replicates by RaxML[91]) showed at least one strongly supported phylogenetic conflict, we also suspect that organellar inheritance has not been strictly maternal in these Corallinales. Two processes - the coalescence of sporelings and the fusion of crustose individuals- may have produced genetic mergers[92]. In addition, phylogeographic analyses indicated that individual Corallinales are good dispersers, as identical sequences of CO1 and *psbA *were found over 1800 to 6300 kms of distance (Figure 3.B). Thallus fragmentation and reattachment on drifting substrates[93], and a quick dispersal of haploid spores by strong currents can introduce individuals in a locality, which generates apparent "barcode gaps", if the incoming individuals are genetically different from the majority of the local population. Yet, at a larger scale, this dispersal of Corallinales results in a continuum of genetic diversity (Figure 2).

Both theories and case-studies indicate that grey-zone related issues are common[51,60] rather than the exception, which too often limits the efficacy of methods based on a single marker to enumerate "what is there" in a pragmatic way. Consistently, the use of a larger number of barcodes[8,27] and of more data is recommended to test when DNA barcoding proposes robust groupings[51,64,94-96], as advocated by the tenants of an integrative taxonomy combining DNA barcoding with other lines of evidence[97,98]. Typically, corroborative data (ecology, morphology, other genes) can help to assign species status to barcode groups. When groups are robust, intra-approach pluralism is - at least - not an issue. Yet, as the sequencing of additional independent barcodes is increasingly recognized a requirement to design robust DNA barcoding analyses, the scope of DNA barcoding will likely expand, and become some sort of multi-marker ... metagenomics. This transition is possible because, although these approaches are rooted in distinct philosophies about species, for most organisms, they largely face similar practical issues as they lack a pragmatic way to define these units.

## Testing the hypothesis

### The highway of strategic networks

To date, metagenomic analyses of multiple random markers seem the most pragmatic recipe to study "what is out there" when informative species taxa cannot be easily identified. However, an inclusive framework is still required to organize vast amounts of molecular data, and to provide information about two other key questions of biodiversity studies: "what is happening?" and "how did this diversity evolve?" Remarkable biodiversity studies have already offered deep biological insights by integrating the results of genetic, taxonomic (using phylotypes) and functional analyses of metagenomic (and metatranscriptomic) datasets, with explicit concerns for the processes maintaining the diversity in communities and environments [31,33,99-102]. For instance, Qu *et al.*[101] unravelled dynamic microbial communities in chicken cecal microbiomes, adjusting to their hosts diet, thanks to mobile DNA elements carrying abundant antibiotic resistances[101]. We will briefly argue that further developments of evolutionary gene networks (EGN) may be a natural follow-up for such studies of integrative metagenomics, be they used to investigate the diversity of one or several environments, or of a set of specimens.

**Figure 4:**
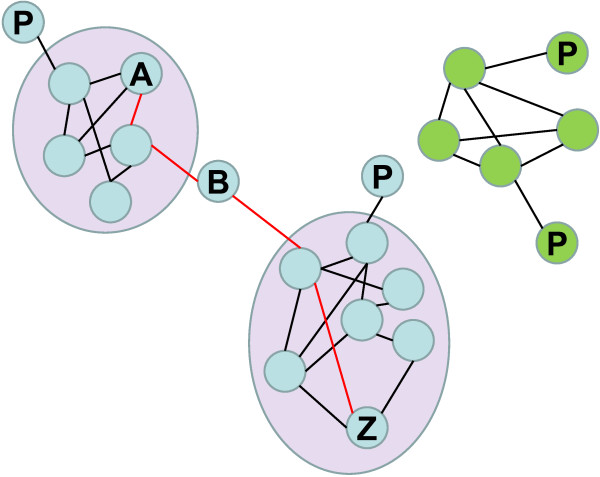
**An example network**. Nodes (circles) are connected by edges (black lines), which may be assigned values or lengths. Blue and green nodes do not share any connections, so they fall into two separate subnetworks (called connected components). Likewise, any two blue nodes are connected by one or more paths. The shortest path between nodes A and Z is displayed in red. Densely connected parts of the network are called modules and are represented in purple here. Some nodes have remarkable topological properties. For example, node B has a high betweenness since it has a high probability of lying in the shortest path between two random nodes. Nodes P, on the opposite, are called peripheral, since they are highly eccentric.

EGN are very inclusive graphs, amenable to specific mathematical investigations (see Additional file 2 for all the related technical terms below), showing both the rarest and the dominant sequences under study. They offer a structured framework to represent and to compare the genetic, functional and processual diversity of multiple datasets in a single analysis. In an EGN, each node corresponds to a sequence (ideally an ORF), with or without taxonomic and functional assignation. Two nodes are connected by edges if their sequences show significant similarity (Figure 4). Edges can be weighted, for instance using the best BLAST score of pairwise comparisons of sequences, so that most similar sequences are closer on the graph. Since not all gene forms resemble one another however, discontinuous variations will structure the graph.

**Figure 5:**
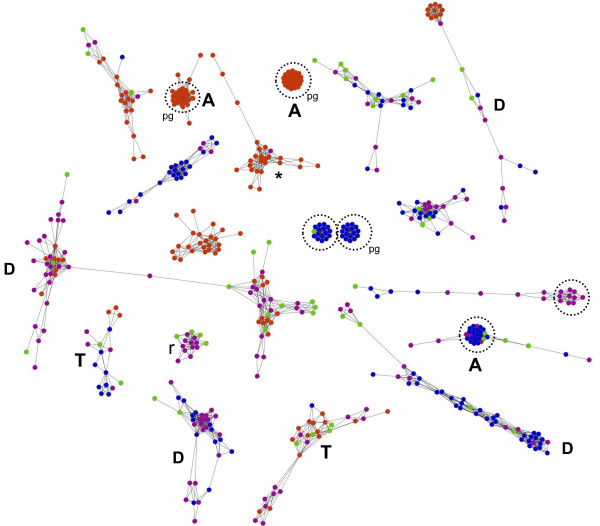
**An inclusive evolutionary gene network **. This graph is a section of an EGN reconstructed using 454 reads from 4 marine environments. Each node represents a genetic sequence. Two nodes are connected by an edge when their corresponding sequences present a significant similarity. All nodes from a given connected component fall into an Operational Gene Family (OGF). Colors correspond to the environment of origin of the sequences, so single coloured OGFs are environment specific. Some OGFs show more genetic variability (indicated by a D), others are highly conserved. T marks OGFs with homologous copies carried on mobile elements. A/R indicates abundant/rare sequences. Circles identify modules, pg indicates when these modules are amenable to studies of population genetics. Topological properties of the connected components, along with the distribution of various colors, are not random. Genetic diversity in the red and blue environments seems complementary, as 77% the connected components separate sequences from these two environments.

An EGN is not fully connected, but comprises multiple subnetworks (connected components) of various sizes and shapes, clustering some sequences together to the exclusion of others. Such connected components define Operational Gene Families (OGF), which organise the data in a molecular space (Figure 5). In practice, these EGNs are easy to reconstruct. Hundreds of thousands of DNA (or proteic) sequences are all BLASTed against each other. The results of these BLASTs (the best BLAST scores between two sequences, their percent of identity, the length over which they align, etc.) are stored in databases. Groups of homologous sequences (the OGF) are then inferred using clustering algorithms (such as the simple linkage algorithm). The BLAST score or the percentage of identity between each pair of sequences is used to weight the corresponding edges. The same procedure can be applied by including sequences of mobile genetic elements in the analyses to figure what OGF are currently mobilized. New samples and sequences can also be very easily added to the analysis.

Observing what and how many OGF are shared (or not) between various samples may be a first step for a biodiversity study. OGF will vary with the threshold retained to define significant similarities, and in that regard OGF definition is just as arbitrary as the decision on where to apply new species names. However, since EGN analysis is inclusive, OGF are defined simultaneously for different samples and environments, and the diversity of different samples can then be compared in one study. The richness and evenness of OGF in a sample, or the complementarity between samples, can be measured with usual diversity indices and methods applied to OGF (Shannon, Pielou, Chao1, ACE, rank-abundance or saturation curves)[7-9]. Furthermore, since EGNs are mathematical objects, their topological properties[103,104] (Figure 4) can be exploited to the benefit of biodiversity studies.

For instance, let's assume that sodium exporters are strongly enriched in marine environments, while potassium exporters are strongly enriched in soils, reflecting the abundance of these ions in these environments[19]. In an EGN comprising sequences from soil and marine samples, OGF with sequences of potassium exporters, and OGF with sequences of sodium exporters, should have more representatives, thus comprise more nodes than average OGF. If in addition novel forms of potassium exporters recently evolved in some soils only (e.g. in farm soils), these new sequences will occupy remarkable -peripheral- positions in OGF with potassium exporters, affecting the very topology of the EGN. Centrality measures, useful for identifying nodes with remarkable positions in a graph can be used to single out such peripheral sequences, since sequences only associated with farm soils will loosely connect with the other potassium exporters sequences. Moreover, the genetic diversity[105] introduced by these new forms of potassium exporters can be quantified by measuring their impact on the diameter of the OGF (the larger its diameter, the more genetically diverse the OGF is). In general, EGNs may thus prove helpful to identify what sequences and gene families play an important functional role, and had their evolution likely impacted by their milieu (Figure 5).

Suppose now that the novel forms of potassium exporters from a given farm soil are all strongly connected in one OGF. In terms of graph theory, they belong to a module[106], which may typically serve to reveal the evolutionary and ecological processes sustaining the diversity in this environment. If the number of organisms in the farm sample was large relatively to the number of sequences obtained, each sequence in the module likely comes from a different organism[107]. Thus, standard population genetics techniques applied on these sequences could provide both refined estimates on the extent of recombination[16] and on the selective pressures acting [108] on potassium exporters sequences in that sample. Similar investigations could be extended to get global estimates, by considering all the sequences from a given sample, falling in all the modules present in the EGN. Finally, global estimates on the processes maintaining the genetic and functional diversity in the samples (e.g. transposition, transfer, molecular regulation and duplication) can also be obtained by counting the relative proportions of OGF with duplicated sequences, homology to transposons[101,109], phages[22,33] or plasmids[110], or harbouring toxin/antitoxin systems[111]. Integration of these various lines of knowledge on bioprocesses and biospecifics in a single EGN might then suggest what evolutionary process affect what OGF (and thus what functions), in what environment (Figure 5).

## Implications of the hypothesis

Biodiversity is far too complex to be adequately addressed by any single approach. For that reason, DNA barcoding approaches and the massive sequencing of multiple markers are obviously not mutually exclusive. However, the latter can comprise the former (while the opposite is not true). Since integrative metagenomics is more inclusive than DNA barcoding, we recommend scientists designing biodiversity studies to use either both approaches as complementary, or for pragmatic reasons - when species problems are observed or expected-, to couple integrative metagenomics with EGNs methods. This latter protocol has the potential to improve the identification and the quantification of biodiversity. It reduces the risk of producing biodiversity estimates with limited explanatory power, biased either by unequal rates of LGT, or difficult to interpret due to (practical) problems caused by type I and type II grey zones. Moreover, it would be easy to include (meta)transcriptomic and (meta)proteomic data in such EGNs, as it would only require the inclusion of such additional molecular sequences in the analyses. The resulting EGNs would then provide a precious framework and useful mathematical tools for studying the almost instantaneous changes in biodiversity, and the immediate catalytic potential of different environments[100]. Relative variations in EGNs, for environmental samples obtained over time or across sites, would thus return relevant indications on the flexibility and resilience of the environment.

## Competing interests

The authors declare that they have no competing interests.

## Authors' contributions

PC & BdR conceived of the study, and collected the specimens. LB & CC carried out the molecular genetic studies. SH and LB conceived of and performed the analyses, and drafted the manuscript, PL & EB conceived of and performed the analyses, and wrote the manuscript. All authors read and approved the final manuscript.

## Reviewers' comments

### Review by William Martin (Institut fuer Botanik III, Heinrich-Heine Universitaet Duesseldorf Universitaetsstr. 1, 40225 Duesseldorf, Germany)

This is a fine paper underscoring the need to take metagenomic data and network approaches into consideration in biodiversity issues. There is no need for major revision in my view, but I came away with three impressions.

Number one, a real life example comparing barcode and "metanet" data for biodiversity investigation, head to head, would perhaps be instructive.

*We agree. We added a new section entitled 'Corallinales as a case-study' to better illustrate the limit of barcode approaches on real datasets, and how this limit could be in part explained by highlighting two evolutionary processes (unequal evolutionary rates in markers and high organismal dispersal range) using two very simple gene networks *(Figure 3A and 3B). *First, using identical settings for the display, a metanet showed that, for the same 206 specimens, CO1 sequences present more divergence than psbA sequences, which means that CO1 had a higher rate of evolution than psbA in these organisms. Second, we used metanets to identify which identical sequences (or nearly identical ones, depending on the threshold selected) were found in geographically different sites, thus testifying of the long distance dispersal of these taxa. We hope that this very simple case study will encourage future metanet analyses of Corallinales (through the sequencing of additional genes from such specimens), and then allow a much finer head to head comparison. We also revised our example of a real environmental metanet *(Figure 5) *to better illustrate modules, environmental specific gene families, abundant families, rare families, transferred families and what parts of the graph singled out markers that are amenable to population genetics analyses*.

Second, in the network analyses, a threshold of sequence similarity has to be introduced; in the paper, the sentence reads: "Two nodes are connected by edges if their sequences show significant similarity". Deciding where to draw that line is just as arbitrary as the decision on where to apply new species names or where to delineate taxon × from taxon y using barcode data. That needs to be said, I think, to be honest that there is no easy way out of these problems and that networks pose new problems of similar nature as the old ones.

*We agree and edited the text to make this point clear. An important difference however with separated DNA barcode analyses and the metanet approach is that metanets are inclusive. Thus the diversity of various datasets can be directly compared, as a same threshold is applied to quantify comparable 'elements' in all of these datasets simultaneously*.

Third, taxonomists often used, and still use, the trusty concept of "discontinuous variation" when it comes to drawing lines. That concept would be useful here, as much of these biodiversity debates concern the question of discontinuity in variation. (Darwin recognized that).

*Discontinuous variation is indeed of great importance, and we now mention it in the revised version of the MS. In metanet analyses, discontinuous variations can be easily identified at two levels. First there are discontinuities between the different Operational Genes Families (OGFs). Second, certain type of discontinuous variation can be unambiguously detected within a OGF using centralities. Typically, OGFs comprising two clusters of sequences only bridged by one intermediate sequence but no other direct connections is an obvious case of discontinuity. These remarkable patterns can be quantified, and their numbers between datasets compared in a single inclusive analysis*.

Some thought could be give to that, or not.

*These are definitely important questions. We thank the referee very much for his helpful comments on these three essential points*.

### Review by Dr. David Williams (nominated by J Peter Gogarten) (Department of Molecular and Cell Biology, University of Connecticut, Storrs, CT 06269-31258, USA)

This article tackles two areas linked by the 'species concept' problem: the ideal of a universally applicable measure of biodiversity across all Domains of life, and a standardised and inclusive way of dealing with the nebulous data from metagenomic surveys. The arguments are clearly presented and I agree with them. Ultimately, EGNs fulfill the authors' billing as a useful addition to the metagenomic toolkit with more potential for integrative analyses.

Many metagenome studies group similar sequences together ('binning') to allow quantitative analyses. Evolutionary Gene Networks (EGNs) place clustered sequences into operational gene families (OGFs) which are analogous to bins but place an emphasis on the diversity and process information within and between these units which I think is a good thing. However, OGFs and lower-level modules are ultimately determined by arbitrary cutoffs in BLAST scores or a chosen clustering method respectively. The authors state there are multiple ways of defining clusters and EGN sub-units. To what extent do biodiversity conclusions vary with different BLAST cutoff scores and module definitions/clustering approaches? Are potential variations great enough for a recommendation towards specific, standardised clustering approaches or cutoff scores?

*Indeed, conclusions may vary with the different BLAST cutoff scores selected: higher cutoff scores will define more stringent OGFs (e.g. OGFs with sequences showing more identity), lower cutoff scores will define looser OGFs (e.g. OGFs with more divergent sequences, such as fused or fissioned sequences, fast evolving sequences, and so on). Importantly, EGNs can then be different as the cutoff changes, especially when the processes sustaining diversity changed over time. As such, EGNs provide a great way to test whether such changes occurred (and for which gene families it occured). That's why we would recommend to explore a range of cutoff scores: from very low ones (e.g. BLAST scores of 1e-5 to study the evolution of biodiversity over the longest time period possible) to very high ones (e.g. BLAST scores of 0 plus 100% of identity between sequences). If the number of OGFs plateaus as a function of these cutoff scores, then one can be confident that the structure and the biodiversity observed in the EGN is robust over time. What is essential however is that the EGNs are both inclusive and grounded in a pragmatic perspective: the questions one wants to address determine what level of cut-off is required. Consequently, it is also important to keep in mind that both high and low cutoff scores have their merits. Consider two extreme cases. First, at low BLAST scores (e.g. of 1e-5), some families will show diverging sequences, but other won't, suggesting that the later have a much more constrained evolution than the former. Second, very high cutoff scores (e.g. BLAST scores of 0 plus 100% of identity) will allow for instance to identify identical sequences dispersed over long geographical distances*.

If rates of evolution across a metagenome vary, is a common BLAST cutoff score across one or more datasets appropriate for inferring the potentially heterogenous processes causing diversity? If inferences of evolutionary processes are to be inferred from network topology, do the authors consider it feasible or desirable to use models of sequence evolution to provide evolutionary distances for weighting edges instead of BLAST scores?

*For some studies, simple EGNs (with homology or BLAST scores) will be very useful and sufficient to improve our knowledge on biodiversity and its evolution. In other cases, it can certainly be desirable to use models of sequence evolution to provide evolutionary distances to weight the edges of EGNs, instead of BLAST scores, in particular when sequences fall in a tight cluster (which means that all of them can be aligned). In general though, developing new evolutionary models (and distances) to generate EGNs with weighted edges that take into account heterogenous evolutionary processes seems a most interesting prospect*.

### Review by Dr. James McInerney (nominated by John Logsdon) (Molecular Evolution and Bioinformatics Unit, Biology Department, NUI Maynooth, Ireland)

I think this is a very interesting manuscript. The authors step back from any particular ecological dataset and consider the approaches that are being taken, their likely outcomes and their potential shortfalls. My own personal opinion is that we will really only begin to do meaningful microbial ecology when sequencing methods are invented that provide us with very long sequences. Some of the results presented here give a glimpse of the kinds of analysis that should be carried out (specifically the homology networks).

*We agree with the referee and thank him very much for his interest in homology networks*.

I agree with the authors that barcoding cannot get us very far in the prokaryotic world and there are serious limitations and serious questions about what is really being addressed when a barcode is derived from an organism.

*We feel that it is a very important point: there are cases where the aims and scopes of DNA barcoding should be critically assessed. Assuming that this approach can be used as an exploratory tool to identify new species (which is the second aim of DNA barcoding, the first being to assign unknown specimens to already recognized species, thanks to a DNA-library of named specimens) will work in every case might lead to misleading results, caused by sampling artefacts and an excessive confidence in the existence of discontinuous variations (assumed rather than tested) in one's dataset. This claim should not be seen as a negative one however, but as a positive incentive for further critical developments in molecular based biodiversity studies (e.g. by promoting critical barcoding analyses, as well as the use of other approaches, when needed)*.

My points are relatively minor and relate to the text and some clarifications I would like to see with certain sentences. I hope this manuscript encourages more discussion of microbial ecology and in particular the methods and what the experiments really mean.

Specific points:

In your abstract you say that you came to your conclusions by evaluating the" two molecular approaches for assessing biodiversity. However, there are more than these two approaches, so perhaps it is best to drop the word 'the'.

*We agree and corrected the sentence*.

on page 3, you write: "Based on their divergences, we propose that an integrative approach aiming at the simultaneous examination (or even better at the integration) of the elements- based, function-based and process-based perspectives in biodiversity studies, thanks to massive sequencing of multiple environmental markers." This sentence does not read properly. Perhaps you did not mean to include the word 'that'?

*We agree and corrected the sentence*.

On page 4, when discussing LGT, you use the sentence "It affects the identification of phylogenetic-species." I presume it is LGT that affects the identification of phylogenetic species (also, I am not sure the hyphen is necessary). Perhaps this sentence can be changed to be more explicit?

*We changed the text accordingly*.

On page 4, you start a paragraph with "The contrast with DNA barcoding [...]" I found this opener difficult to interpret. What is being contrasted with what?

*We revised that part of the MS, including additional sub-titles to clarify what was contrasted with what*.

On page 5, the sentence "First, when the various secondary properties defining the groups arose at different times in the process of speciation, the proposed groupings are nested (Type I grey zone, Figure 1D)" changes tense and makes it difficult to read.

*We changed the text accordingly*.

## Supplementary Material

Additional file 1 **Corallinales dataset**. Click here for file

Additional file 2 **Glossary**. Click here for file
