# Supplementary material for: Some considerations for analyzing biodiversity using integrative metagenomics and gene networks
Source: Biol Direct. 2010 Jul 30;5:47. doi: 10.1186/1745-6150-5-47 (PMC2921367; doi:10.1186/1745-6150-5-47)
Supplement: Additional file 2 — Glossary. [file 1745-6150-5-47-S2.DOC]

**Additional file 2: Glossary**

**Network** : a network is an informal description of a set of objects that share connections. Formally, they are represented as graphs where objects are called vertices (or nodes) and connections are called edges.

**ORF** : Open Reading Frame, a part of a genome that can potentially encode a protein.

**Edge** (weighted or not) : a connection between two vertices of a graph. Edges can be assigned a numerical value, which is then called a weight. If not, edge weights are arbitrarily set to 1.

**Connected component** : two vertices are in the same connected component if and only if there is a path between them. Practically, connected components are the isolated subnetworks of a larger network.

**Operational gene family** : sequences that belong to a given connected component in a evolutionary gene network.

**Shortest path**: a path is a set of adjacent edges that connect two nodes. Its length is defined as the sum of the weights of these edges. If multiple paths are possible between two nodes, the shortest path is the path of minimal length.

**Diameter** : the diameter of a connected component is the maximum of the shortest path lengths between all pairs of vertices.

**Centrality** : a numerical value assigned to each vertex, usually describing its topological properties in the network. Common centralities include the degree of a vertex (i.e. the numbers of edges it belongs to) or the betweenness of a vertex (i.e. its probability to lie on the shortest path between two vertices of the network).

**Periphery** : the eccentricity of a node is defined as the maximum length of the shortest path from that node to any other one. Usually, vertices that belong to one edge only show a high eccentricity and are said to be peripheral, since they appear on the outside of edge-weighted displays of graphs.

**Module** : a subset of vertices that are more connected between them than with the rest of the network. Much like clustering, there are many ways of defining modules.
